# Septate uterus with hypoplastic left adnexa with cervical duplication and longitudinal vaginal septum: Rare Mullerian anomaly

**DOI:** 10.4103/0974-1208.69331

**Published:** 2010

**Authors:** Aruna Nigam, Manju Puri, Shubha Sagar Trivedi, Barenya Chattopadhyay

**Affiliations:** Department of Obstetrics and Gynaecology, Lady Hardinge Medical College, New Delhi, India

**Keywords:** Mullerian anomalies, septate uterus, vaginal septum

## Abstract

A large analysis of all the studies in the period from 1950 to 2007 suggests that the prevalence of congenital uterine anomalies in the general population is 6.7%; and in the infertile population, 7.3%. We report a rare case of unilateral hypoplastic fallopian tube and ovary with septate uterus, cervical duplication, longitudinal vaginal septum. To the best of our knowledge, this is the first report of such a congregation of anomalies.

## INTRODUCTION

A large analysis of all the studies in the period from 1950 to 2007 suggests that the prevalence of congenital uterine anomalies in the general population is 6.7%; and in the infertile population, 7.3%.[1] The rate of mullerian anomalies lies between 3% and 25% in women with a history of repeated pregnancy loss.[2,3] We report a rare congregation of mullerian anomalies consisting of unilateral hypoplastic fallopian tube and ovary with septate uterus, cervical duplication, longitudinal vaginal septum. To the best of our knowledge, this is the first such case report. Although simultaneous occurrence of septate uterus, cervical duplication and longitudinal septum has been reported in the literature, yet association of hypoplastic left fallopian tube and ovary renders the case unique.

## CASE REPORT

**Figure 1:**
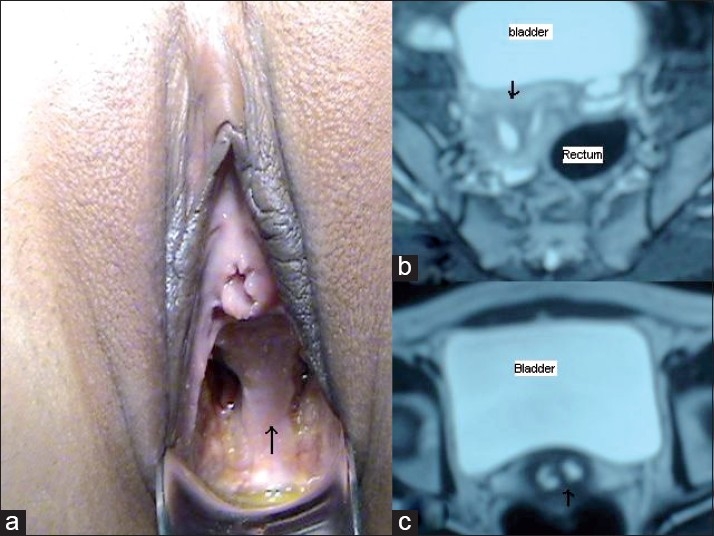
(a) Thick longitudinal vaginal septum indicated by arrow, (b) MRI showing septate uterus indicated by arrow, (c) MRI showing duplicated cervix indicated by arrow

**Figure 2:**
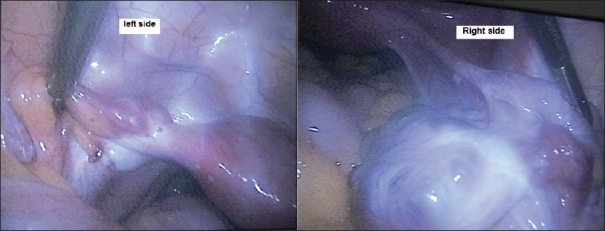
Small left tube and left ovary (left window), normal right tube and normal ovary (right window)

A 30-year-old woman presented with a complaint of dyspareunia and primary infertility. She had regular menstrual cycles without dysmenorrhea. Her general and systemic examinations were normal. On speculum examination, a thick midline longitudinal vaginal septum was seen in the upper two thirds of the vagina [Figure 1a]. Two cervices were visualized on either side of the septum. On vaginal examination, uterus was normal in size, anteverted, mobile; and bilateral fornices, clear. Ultrasound showed a bicornuate uterus and normal ovarian stroma and follicles, although the left ovary was smaller in size (left, 1.4×1.1×0.4 cm; right, 3.2×2.3×1 cm). MRI scan showed completely septate uterus with duplication of cervix and vaginal septum [Figures 1b and c]. No renal tract anomalies were visualized on the MRI scan. On hysteroscopy, 2 separate uterine cavities were visualized with normal cornua in either cavity. On laparoscopy, uterus was normal in size and shape with a single fundus, which was slightly broad. Right tube and ovaries were normal. The left fallopian tube was normal in origin and diameter but only 4 cm in length with very small left ovary, i.e., less than half the size of the right one [Figure 2]. On chromopertubation, free spillage of dye was present on the right side and no spillage on the left side. Vaginal septum resection was done. The patient has been on follow-up for a period of 6 months but has not conceived yet. Such patients can conceive naturally at times.

## DISCUSSION

The congregation of septate uterus, cervical duplication, partial longitudinal vaginal septum, left fallopian tube and ovarian hypoplasia occurred as distinct events during genital tract formation in the early weeks of organogenesis. The events leading to these findings are relatively not well understood separately, and why they all occurred at the same time is a matter of pure assumption. It has been proposed that a unilateral defect in the development of the urogenital ridge could have profound effect on the future gonad which would lie upon it. Due to the physical proximity of the distal fallopian tube to that region, one could speculate that the improper development of the urogenital ridge could also affect development of the fallopian tube in that region.

The genesis of septate uterus, cervical duplication and partial longitudinal vaginal septum cannot be explained by classic hypothesis of unidirectional (caudal to cranial) fusion of mullerian ducts as detailed by Crossby and Hill in 1962. Hundley *et al*.[4] described in a similar case report how this anomaly could not follow the classic progression. This anomaly demonstrates that the mullerian duct fusion could start at the isthmic area and then travel in both caudal and cranial manner as suggested by the alternative embryologic theory of Muller. Fusion might occur in bidirectional manner, or the duplicated cervix and vagina septum might suggest a lack of caudal fusion; whereas septate uterus superiorly might point to successful fusion but lack of resorption superiorly. Many of these patients had thick uterine septa which contained substantial myometrial tissue.

Suh and Kalan have reported a similar case of fallopian tube hypoplasia with ipsilateral ovarian agenesis; but in their case, there was no cervical duplication and septate vagina.[5] Fallopian tube hypoplasia and unilateral ovarian agenesis appear in the literature mostly as case reports. Asymptomatic segmental torsion of the uterine tube and/ or ovarian pedicle may occur for uncertain reasons during adulthood, in childhood or even during the fetal stages, and consequently, torsion may give rise to necrosis and autoamputation. Secondly, the absence of these organs may be congenital, associated with developmental alterations of the mesonephric and paramesonephric ducts.[6] However, total agenesis was not present in this case. It is postulated that the mullerian anomalies appeared distinctly in this patient and so did the developmental anomaly of the urogenital ridge, the latter resulting in ovarian hypoplasia causing secondarily the hypoplasia of the adjoining fallopian tube. Further, it is postulated that both the developmental anomalies occurred simultaneously in this patient, giving this case its uniqueness. Failure of dye spillage on chromotubation from the left tube could possibly be a part of the developmental anomalies or a consequence of occult tubal infection.

MRI has been the “gold standard” for categorizing uterine anomalies because of its 98% to 100% accuracy.[7] T-2–weighted images best discern these anomalies. Septate uterus is characterized on MRI by the presence of a uterine fundus with relatively normal outer contour and a uterine cavity with normal intercornual distance, but separated by a septum. However, Wai *et al*.[8] have emphasized the utility of laparoscopy-assisted hysteroscopy in these cases for diagnostic purposes. Moreover, for the diagnosis of fallopian tube anomalies, laparoscopy is the gold standard.

As this mullerian anomaly is fairly unique, without data on long-term outcomes, it is uncertain what type of treatment is necessary to maintain the best reproductive capacity. However, if this behaves more like class 5 type of uterine anomalies, then there might be evidence that demonstrates some benefit in hysteroscopic resection of septa and consequent decrease in rate of miscarriage. Recent evidence is emerging that resection of the uterine septum and the cervical septum improves the reproductive performance in this anomaly.[9] Among the mullerian anomalies, unicornuate uterus has the poorest reproductive outcomes.[10] It is our hope, however, that the reporting of this case along with the existing literature will help to shed some light on female reproductive development and the anomalies which go along with it. Precise diagnosis and reports of such cases are important not only for the benefit of treatment but also to consolidate the embryologic concept.
